# Cerebral CIC-NUTM1 rearrangement sarcoma- case report and review of the literature

**DOI:** 10.3389/fonc.2025.1519335

**Published:** 2025-04-04

**Authors:** Wei Jin, Ke Dong, Fan Yang, Jianning Zhang, Gang Cheng

**Affiliations:** ^1^ Department of Pathology, Chinese People's Liberation (PLA) General Hospital, Beijing, China; ^2^ South China University of Technology, Guangzhou, Guangdong, China; ^3^ Department of Neurosurgery, Chinese PLA General Hospital, Beijing, China

**Keywords:** CIC-NUTM1, CIC rearrangement sarcoma, brain, Ewing sarcoma, Ewing-like sarcoma

## Abstract

**Background:**

Ewing sarcoma and Ewing-like sarcoma are both highly aggressive small round cell sarcomas, while CIC rearranged sarcoma (CRS), the most common specific type of Ewing-like sarcoma, exhibits a more aggressive course than Ewing sarcoma and represents a distinct family of sarcomas. NUT midline carcinoma family member 1 (NUTM1) is a characteristic fusion gene of NUT midline carcinoma. In this paper, the intracranial tumor CIC is mainly fused with NUTM1, which is considered to be a molecular variant of CIC sarcoma.

**Case presentation:**

We report a 9 years old female patient diagnosed with CIC rearrangement sarcoma with CIC-NUTM1 gene rearrangement and PMS2 frameshift mutation, WHO grade 4. We treated the patient with surgical resection. Due to the poor postoperative condition of the patient, coupled with the inherently poor prognosis of CIC-NUTM1 sarcoma, the patient ultimately had a short survival time and the treatment outcome was not satisfactory.

**Conclusion:**

We experienced a rare case of an intracranial tumor with CIC-NUTM1 fusion and a PMS2 frameshift mutation. Due to the small sample size, rapid progression, and poor prognosis associated with this type of tumor, it is essential to enhance understanding and diagnosis of this type of sarcoma.

## Introduction

The Capicua transcriptional repressor (CIC) rearranged sarcoma is a high-grade, undifferentiated sarcoma that occurs in the neural axis, characterized by recurrent translocations involving the CIC. There are several partner genes related to the CIC gene in CIC rearranged sarcoma, including DUX4, FOXO4, LEUTX, NUTM1, and NUTM2A, with the most common fusion type being CIC-DUX4. In this paper, the intracranial tumor CIC is primarily fused with NUTM1 (1), which is considered to be a molecular variant of CIC sarcoma. When the two fuse, the methylome and transcriptome profiles align more closely with CIC-DUX4 sarcoma rather than NUT carcinoma. This fusion was first reported by Watson et al. in 2018, demonstrating an aggressive biological behavior, with a histomorphology ranging from round to epithelioid cells and a scattered rhabdoid morphology, exhibiting unique immunohistochemical and genetic phenotypes. So far, a total of 13 cases (2, 4–6) have been reported (6 cases in 2019 and 3 cases in 2022, with the rest being individual case reports), including 7 pediatric cases with a male-to-female ratio of 7:6, aged 2-61 years (average age 24.3 years); Tumor diameter ranged from 2.0-12.5cm (average 6.8cm). The primary tumor site for deep soft tissue (n = 3), vertebral body (n = 3), skull (n = 2), extradural spinal cord (n = 2), brain (n = 1), kidney (n = 1), lung (n = 1); 8 out of 11 cases (72.7%) involved the vertebral body (5 cases) or the skull base (3 cases), showing local invasion of bone and surrounding soft tissue, only 2 cases occurred in the temporal and occipital regions and involved the brain. In this case, the patient is a 9-year-old girl with a tumor diameter of approximately 6.6 cm, located mainly on the right side of the medulla oblongata, right anteriorly, right cerebellopontine angle, right jugular foramen, right carotid sheath, right parapharyngeal space, involving the skull with visible bone destruction, causing compression and deformation of the brainstem and fourth ventricle.

## Case presentation

**Figure 1:**
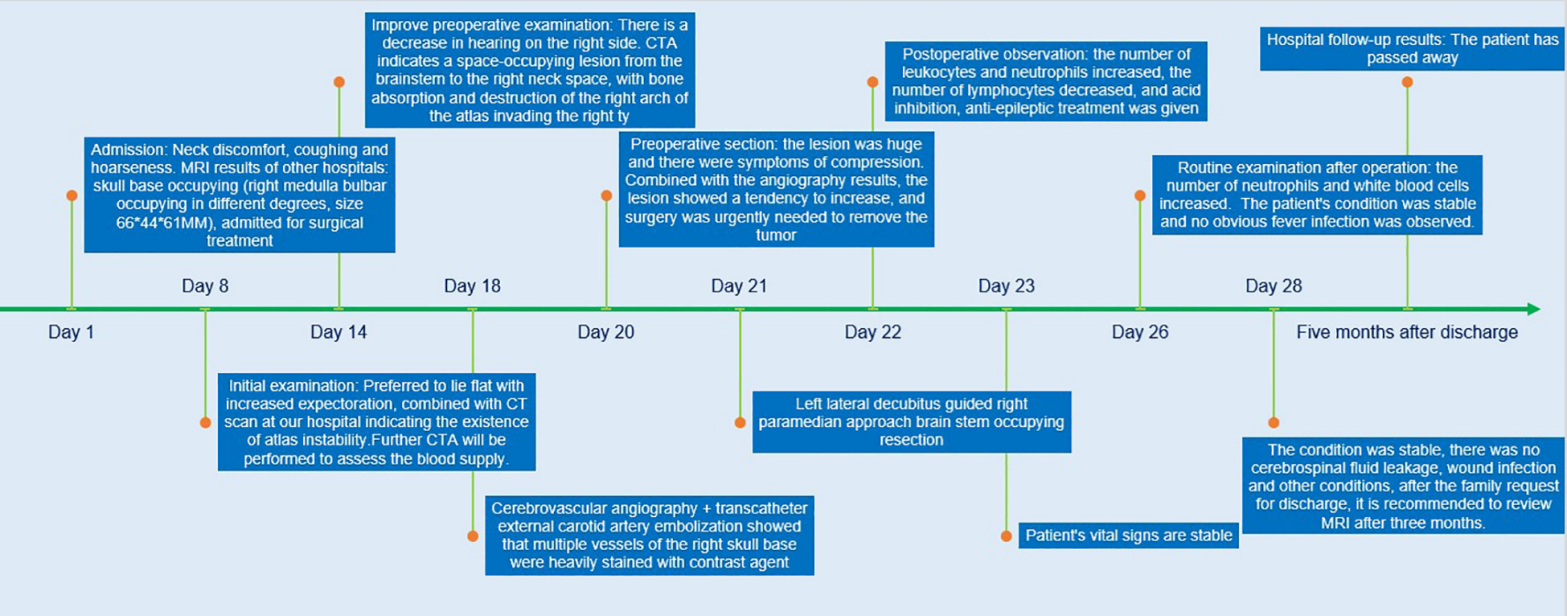
Treatment timeline.

**Figure 2:**
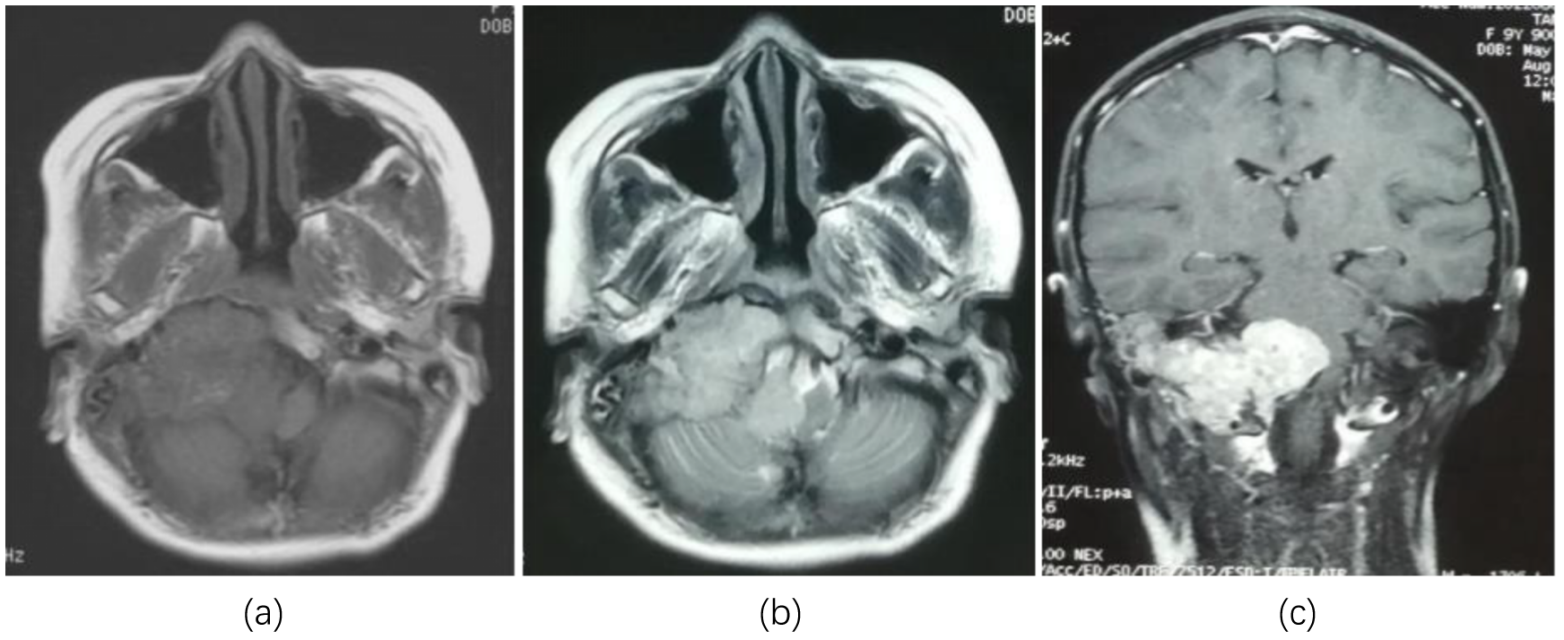
Head MRI showed lesions in the right and right anterior medulla oblongata, right slope area, right jugular foramen, right carotid sheath, and right parapharyngeal space occupying lesions. **(a)** Cross section T1WI scan shows a slightly equal length signal; **(b)** cross section T2WI scan showed uneven and slightly long signal; **(c)** Cross-sectional enhanced scanning showed obvious enhancement.

A 9-year-old female patient suddenly developed neck discomfort without obvious causes before 2 months ago, with tight neck plate, cough, hoarseness, and general fatigue in the past 1 month (The treatment timeline after admission is shown in **Figure 1**). The existing head MRI results suggest a mass in the right side of the medulla oblongata, right anteriorly, right cerebellopontine angle, right jugular foramen, right carotid sheath, and right parapharyngeal space. Physical examination revealed positive difficulty in closing the eyes. Head MRI plain scan + enhancement was performed on the patient in our hospital, and the results showed (see **Figure 2**; a. Cross section T1WI scan shows a slightly equal length signal; b. cross section T2WI scan showed uneven and slightly long signal; c. Cross-sectional enhanced scanning showed obvious enhancement.): A slightly longer T1 T2 signal, such as irregular mass and mixed mass, was visible on the right and right anterior part of the medulla oblongata, the right slope area, the right jugular foramina, the right carotid sheath, and the right parapharyngeal space. The boundary was clear, and the size was about 6.6x6.1x4.4cm. The corresponding skull showed bone destruction, compression and deformation of the brainstem and the fourth ventricle, which showed obvious enhancement after enhanced scanning.

We performed surgical resection of the tumor, intraoperatively separating the fascia and muscles along the nuchal line, exposing the occipital squama and posterior arch of the atlas. Grayish-red, well-vascularized tumor tissue was visible, eroding the dura mater without clear boundaries.

**Figure 3:**
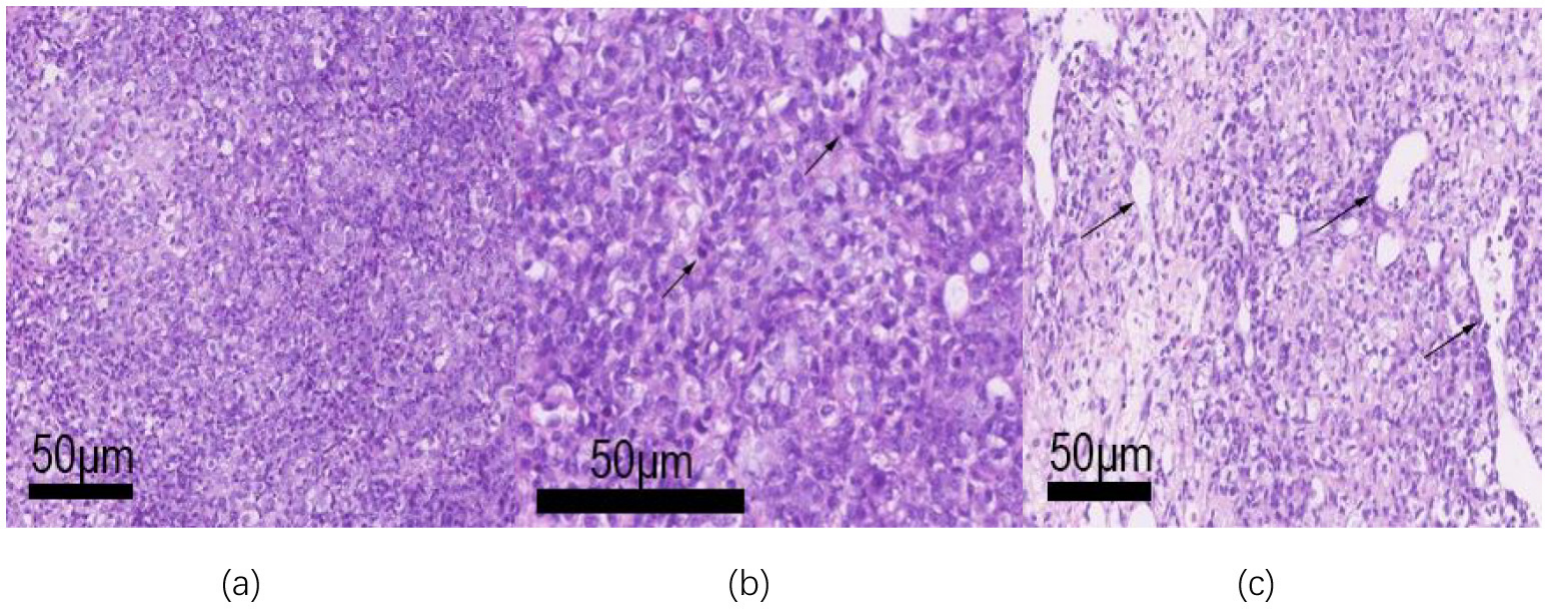
The tumor tissue was observed under a microscope. **(a)** The tumor is distributed in a lamellar, dense, and focal epithelioid changes (low magnification); **(b)** Tumor cell atypia is obvious, nucleoli can be seen, and the mitotic image is easy to see (arrow, medium magnification); **(c)** The tumor cells are sparse in some areas, and slit-like blood vessels can be seen (medium magnification).

**Figure 4:**
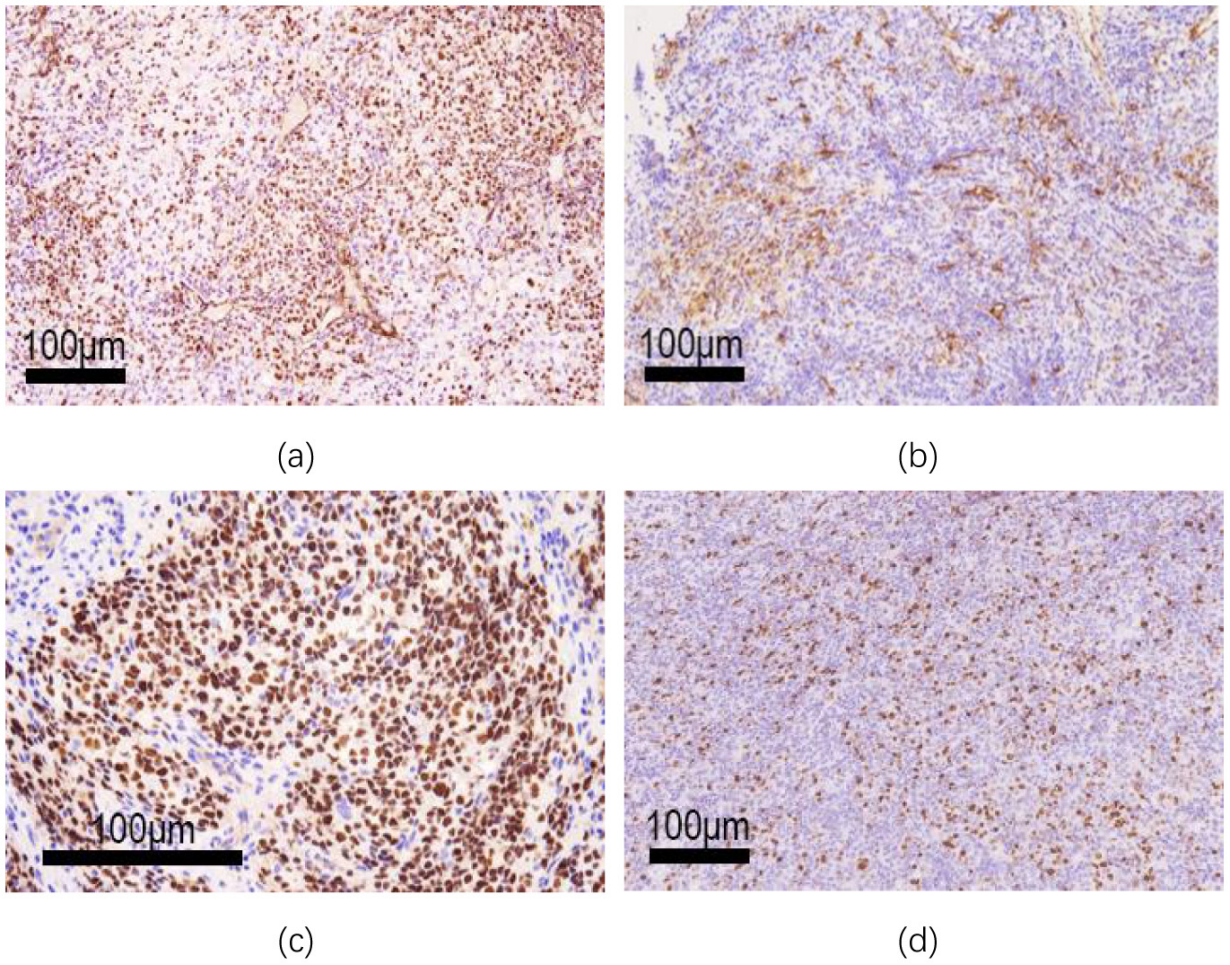
Histochemical and immunohistochemical staining (EnVision two-step method). **(a)** WT-1 immunohistochemical staining of tumor cells was positive (low magnification); **(b)** Focal positive CD99 staining (low magnification); **(c)** Nut immunohistochemical staining showed strong nuclear positive (medium magnification). **(d)** ki-67 labeling index up to 30% (low magnification).

Microscopically, the tumor exhibits a sheet-like distribution, dense with focal epithelioid changes (see **Figure 3a**). The tumor cells are round or oval-shaped, with visible nucleoli, significant cellular pleomorphism, and readily identifiable mitotic figures (see **Figure 3b**). In some areas, tumor cells are sparse, with slit-like blood vessels visible (see **Figure 3c**). Immunohistochemical examination shows positive expression of WT-1 (see **Figure 4a**), CD99 (see **Figure 4b**), NUT (see **Figure 4c**), Ki67 proliferation index of 30% (see **Figure 4d**), Vimentin, FLI-1, Bcl-2, CD56, STAT6, INI-1, BRG-1, with focal positivity for Syn, SMA, and BCOR. Negative expression is observed for GFAP, S-100, EMA, NeuN, SATB2, NSE, NKX2.2, Desmin, CK, Myo-D1, NF, AE1/AE3, CD34, among others. (a. Positive immunohistochemical staining for WT-1 in tumor cells (low magnification); b. Focal positive staining for CD99 (low magnification); c. Strong nuclear positivity in NUT immunohistochemical staining (medium magnification); d. Ki-67 labeling index reaching 30% (low magnification)).

The molecular pathology results show rearrangements of the CIC and NUTM1 genes, as well as a frameshift mutation in PMS2. No mutations were detected in IDH1/IDH2, ATRX, BRAF, P53, H3F3A, or the TERT promoter.

Combining the above test results with morphological, molecular, and immunohistochemical examinations, the diagnosis is a small round cell malignant tumor, consistent with CIC-NUTM1 gene rearrangement and concurrent PMS2 frameshift mutation, classified as WHO Grade 4.

## Discussion

We encountered a rare case of CIC-NUTM1 gene rearrangement. Although the clinical and pathological features of CIC-NUTM1 rearranged sarcomas exhibit morphological characteristics highly similar to those of CIC sarcomas, they cannot be distinguished from NUT carcinoma, showing some overlap. Le Loarer et al. (2) conducted morphological and immunohistochemical analysis of 6 cases of CIC-NUTM1 rearranged sarcoma, and concluded that CIC-DUX4 fusion transcripts induced up-regulation of multiple tumor enhancer activator 3 (PEA3/ETV4/E1AF) family genes, which could express ETV4 and WT1. All CIC-NUTM1 sarcomas except one expressed ETV4, while NUT cancer did not. WT1 appears to be expressed patchy in CIC-NUTM1 tumors, whereas it is expressed in only 2 CIC-DUX4 fusion tumors, including ETV4 negative cases. It is believed that ETV4 positive is more likely to diagnose CIC-NUTM1 sarcoma than NUT carcinoma. At the same time, it was found that both the expression and distribution patterns of NUT immunohistochemistry appeared to be more diffuse and uniform, rather than the light-stained blotchy pattern typical of NUT cancer. At present, the number of cases is small and the significance of this difference is not clear, and larger samples are needed to further elucidate. In a study of 40 patients with CIC rearrangement sarcoma, Hung et al. (3) found that 95% of these tumors and 19% of other round cell tumors (excluding Ewing sarcoma) were immunopositive for WT1, and Calretinin, ERG, FLI1, and TLE1 were also expressed positively in these tumors. The expression of calretinin to varying degrees was observed in about 3/4 of CIC sarcomas, emphasizing that ETV4 has a positive expression rate of 70% in CIC-NUTM1 sarcomas, which is inconsistent with classical CIC sarcomas (positive rate of up to 100%). The results suggest that WT1, calretinin and ETV4 may be useful markers for the differentiation of CIC rearrangement sarcoma and other small round cell tumors. In this study, the tumor expressed WT1 focally, and NUT protein was diffusely and uniformly deeply stained, consistent with the reported literature. In summary, before the absence of molecular and methylation profile confirmation, the microscopic morphology combined with WT1, ETV4, NUT and other immunohistochemical staining is helpful for the diagnosis and differential diagnosis of this tumor.

Besides the gene rearrangement, a PMS p. R578Afs heterozygous mutation appeared as a clear pathogenic gene. Subsequently, we repeated mismatch repair protein (MMR) staining and fluorescence capillary electrophoresis (microsatellite instability, MSI) testing, both of which showed microsatellite stability (MSS), inconsistent with the molecular tests mentioned above. The possible reason is that the mismatch repair system (MMR) functions as a dimer of the proteins encoded by MLH1, MSH2, MSH6, and PMS2 to maintain genome stability when DNA mismatches occur during replication or recombination. MLH1 and MSH2 are essential partners (dominant proteins) that can form heterodimers with other MMR proteins, including MSH3, MLH3, and PMS1, where MSH6 can be replaced by MSH3, and PMS2 can be replaced by PMS1 or MLH3. Therefore, even though a clear heterozygous mutation in PMS2 is detected at the molecular level, there might not be abnormal protein expression at the protein level. Some inherited mutations of the PMS2 gene are associated with the risk of Lynch syndrome (LS); a study in 2023 (7) reported 95 cases of sarcoma-associated Lynch syndrome, with soft tissue sarcomas accounting for 93%, and PMS2 mutations only accounting for 5%. In this study, PMS2 was identified as the pathogenic gene, and there was no family history on follow-up. We speculate that the patient may be the initial case of Lynch syndrome (i.e., the proband) and that the occurrence of the tumor due to CIC-NUTM1 gene rearrangement may not be directly related, potentially operating in separate pathogenic pathways, with the former not yet manifesting clinically.

The accurate diagnosis of CIC-NUTM1 sarcoma is of significant clinical importance due to its poor prognosis. Thirteen reported cases with follow-up data showed a total survival period of 7-37 months, with 8 patients succumbing to the disease. Compared to Ewing sarcoma, it exhibits stronger invasiveness and a worse prognosis than classical CIC sarcomas. In terms of treatment (8), most patients undergo surgical resection combined with chemotherapy (similar to the treatment regimen for Ewing sarcoma patients), neoadjuvant or adjuvant therapy, and adjuvant radiotherapy. Some literature (9) suggests that administering craniospinal and local radiotherapy concurrently results in better overall survival than craniospinal irradiation alone. The patient in this study passed away within 5 months of diagnosis, and due to poor postoperative condition, no further treatment was administered, contributing to the shorter survival period.

## Conclusion

CIC rearranged sarcoma is a high-grade tumor that occurs in the neural axis, with multiple fusion partners. Its clinical presentation and imaging features are nonspecific, but it has unique immunohistochemical and genetic characteristics. The tumor’s location and histological morphology make it easily confused with NUT carcinoma. Therefore, ETV4 expression and the expression and distribution pattern of NUT protein can serve as alternative markers for screening this type of tumor, but ultimately, FISH and/or RNA sequencing, as well as methylation testing, are the gold standard for diagnosis. Currently, there are few cases of this type of sarcoma, it progresses rapidly, has a poor prognosis, and there is a need to enhance awareness and diagnosis of this disease.

## Data Availability

The original contributions presented in the study are included in the article/supplementary material. Further inquiries can be directed to the corresponding authors.
